# Renal involvement in patients with COVID-19

**DOI:** 10.6061/clinics/2020/e2194

**Published:** 2020-10-21

**Authors:** Márcia F. Arantes, Camila E. Rodrigues, Victor F. Seabra, Paulo R.G. Lins, Bernardo V. Reichert, Gabriel T.M. Sales, Igor Smolentzov, Carla P.S. Cabrera, Lúcia Andrade

**Affiliations:** Divisao de Nefrologia, Hospital das Cl?nicas (HCFMUSP), Faculdade de Medicina, Universidade de Sao Paulo, SP, BR

Individuals who are infected with the severe acute respiratory syndrome coronavirus 2 (SARS-CoV-2) and develop coronavirus disease 2019 (COVID-19) are also at risk of developing acute kidney injury (AKI) (1), although the exact incidence of AKI in the context of COVID-19 is unknown. A recently published study evaluated >1,000 COVID-19 cases in China and found that the prevalence of AKI among those patients was 1.6% (2). Another study conducted at a teaching hospital in the Chinese city of Wuhan assessed 701 patients with COVID-19 and reported that the overall prevalence of COVID-19-induced AKI was 3.2% (3). Another interesting nephrological finding was that, at admission, 43.9% of the patients had proteinuria and 26.7% had hematuria. The authors also found that the risk of in-hospital death was significantly higher in patients with kidney disease than in those without such disease.

In a single-center retrospective study conducted in China, data from 333 hospitalized patients with COVID-19 pneumonia showed that 75.4% had abnormal urine dipstick test results or AKI (4). The authors found that overall mortality was higher among patients with renal involvement than among those with no renal involvement (11.2% *vs.* 1.2%). Another single-center retrospective study conducted in China showed that AKI occurred in 15 (29%) of the 52 evaluated critically ill adult patients (5). In a multicenter study, Zhou et al. evaluated 191 adult inpatients with COVID-19 and reported that 28 (15%) had AKI (6). The Italian National Institute of Health reported that within a population of 2,000 patients infected with SARS-CoV-2, the prevalence of AKI was 27.8% (7). In a large cohort of COVID-19 subjects (over 2,000 patients) in New York, 22.2% had AKI and 3.2% required kidney replacement therapy (8).

The mechanisms underlying kidney injury in patients infected with coronaviruses have been studied since the SARS-CoV epidemic. In a study conducted in Hong Kong in 2003, seven patients with SARS who developed AKI and later died underwent postmortem histopathological analysis; significant acute tubular necrosis was found in all cases but no viral particles were found in the renal tissue (9). The authors concluded that the mechanism of AKI in patients is probably multifactorial, including antibiotic interstitial nephritis, acute tubular necrosis due to ischemia, and sepsis due to secondary infections. It is important to note that the study did not exclude the possibility of direct renal injury by coronavirus. The results could have been influenced by the use of polymerase chain reaction to detect viral particles in the urine of the patients and by the fact that the ultrastructural analysis was impaired by the autolysis of renal parenchyma cells.

In another study conducted in Wuhan, Diao et al. performed histological and immunohistochemical analyses to identify the SARS-CoV-2 nucleocapsid antigen, markers of cellular immunity, and complements in renal tissues collected postmortem from six patients diagnosed with COVID-19 and AKI and with unfavorable outcomes (10).

In addition to the extensive acute tubular necrosis previously observed in patients with SARS-CoV infection, the authors identified the accumulation of the nucleocapsid protein antigen in the tubules, infiltration of CD68+ macrophages, and deposition of C5b-9, indicating that the virus is capable of infecting renal tubular cells, causing direct and indirect injury through the activity of macrophages and the complement system. In an autopsy study of a patient with COVID-19 and AKI, Farkash et al. identified intracellular viral arrays in the proximal tubular epithelial cells on electron microscopy, findings that are consistent with direct infection of the kidney with SARS-CoV-2 (11). Su et al. also identified coronavirus-like particles with distinctive spikes in the cytoplasm of the proximal and distal tubular epithelium as well as in the podocytes (12).

The mechanism underlying AKI in COVID-19 includes a maladaptive response of the immune system that induces a cytokine storm and could be responsible for the systemic inflammatory response syndrome-induced AKI (13). Patients with COVID-19 in the intensive care unit (ICU) occasionally require mechanical ventilation, vasopressor drugs, and nephrotoxic drugs, all of which can aggravate AKI.

It is well known that COVID-19 can induce thrombotic events. In the postmortem histopathological analysis of kidney tissue in patients who died from COVID-19, Su et al. observed erythrocyte aggregates obstructing peritubular capillaries and segmental fibrin thrombi in the glomeruli (12).

Rhabdomyolysis is a common finding in COVID-19. It can lead to elevated serum creatinine phosphokinase levels (more than five times the upper limit of normal) and could be another important factor for the development of AKI (4,14). Furthermore, hemosiderin granules have been observed in the lumina of tubular cells in patients with COVID-19 (12).

Angiotensin-converting enzyme 2 (ACE 2) and members of the serine protease family, which are essential for a virus to bind to the host cells, are highly expressed in podocytes and tubular epithelial cells. Therefore, COVID-19 can also cause hematuria and proteinuria, further supporting the idea that the SARS-CoV-2 shows tropism in the kidney (12,15). ACE 2 receptors have been found to play an important role in the development of severe acute respiratory SARS-CoV-2, and thus concerns about the use of renin angiotensin system (RAS) blockers for COVID-19 patients have been raised. Two important studies in Milan and New York showed no evidence of increased risk of severe COVID-19 with the use of RAS blockers (16,17). An experimental study also demonstrated that RAS blockers did not result in an increase in ACE 2 levels in kidney and lung epithelia (18).

There is currently no specific treatment for infection with SARS-CoV-2. Although various drugs are being investigated in clinical trials, the management of COVID-19 continues to be mainly supportive, and a significant number of patients require ICU admission (15).

For patients with COVID-19-induced AKI, renal replacement therapy might be necessary (19). Among ICU patients with COVID-19-induced AKI in Seattle, Washington, 5% required dialysis, typically 2 weeks after the onset of symptoms (20). In a recent meta-analysis of three studies evaluating ICU patients with COVID-19-induced AKI, the proportion of patients requiring renal replacement therapy ranged from 5.6% to 23.1%, with a pooled incidence of 13% (21).

There is still insufficient scientific evidence to support the superiority of one method of dialysis over any other (13). We recommend selecting the type of dialysis according to the severity of the disease, considering the availability of resources and experts in the health care facility in question. In the months of May and June 2020, we performed 704 and 1,235 hemodialysis sessions, respectively, for ICU patients at our facility. Approximately 27% of the sessions have been of the continuous venovenous type (hemodialysis, hemodiafiltration, or hemofiltration).

The choice between continuous and intermittent dialysis depends on the traditional indications (hemodynamic stability, cerebral edema, or the need to remove fluid overload).

We are still learning about renal abnormalities in COVID-19 patients. We hope that we will soon be able identify the best treatment and develop an effective vaccine for this devastating disease.

## AUTHOR CONTRIBUTIONS

Arantes MF was responsible for the editorial idea and conception, manuscript drafting and final approval of the version to be published.Rodrigues CE, Seabra VF, Seabra VF, Reichert BV, Sales GTM, Smolentzov I and Cabrera CPS were responsible for critical review of the manuscript, intellectual content, and final approval of the version to be published. Andrade L was responsible for mentoring, critical review, and final approval of the version to be published.

## AUTHOR CONTRIBUTIONS

Arantes MF was responsible for the editorial idea and conception, manuscript drafting and final approval of the version to be published. Rodrigues CE, Seabra VF, Seabra VF, Reichert BV, Sales GTM, Smolentzov I and Cabrera CPS were responsible for critical review of the manuscript, intellectual content, and final approval of the version to be published. Andrade L was responsible for mentoring, critical review, and final approval of the version to be published.
